# A Comparison of Machine Learning Tools That Model the Splitting Tensile Strength of Self-Compacting Recycled Aggregate Concrete

**DOI:** 10.3390/ma15124164

**Published:** 2022-06-12

**Authors:** Jesús de-Prado-Gil, Covadonga Palencia, P. Jagadesh, Rebeca Martínez-García

**Affiliations:** 1 Department of Applied Physics, Campus of Vegazana s/n, University of León, 24071 León, Spain; c.palencia@unileon.es; 2 Department of Civil Engineering, Coimbatore Institute of Technology, Coimbatore 641014, Tamil Nadu, India; jaga.86@gmail.com; 3 Department of Mining Technology, Topography and Structures, Campus de Vegazana s/n, University of León, 24071 León, Spain

**Keywords:** machine learning, splitting tensile strength, self-compacting concrete, recycled aggregates, prediction

## Abstract

Several types of research currently use machine learning (ML) methods to estimate the mechanical characteristics of concrete. This study aimed to compare the capacities of four ML methods: eXtreme gradient boosting (XG Boost), gradient boosting (GB), Cat boosting (CB), and extra trees regressor (ETR), to predict the splitting tensile strength of 28-day-old self-compacting concrete (SCC) made from recycled aggregates (RA), using data obtained from the literature. A database of 381 samples from literature published in scientific journals was used to develop the models. The samples were randomly divided into three sets: training, validation, and test, with each having 267 (70%), 57 (15%), and 57 (15%) samples, respectively. The coefficient of determination (R^2^), root mean square error (RMSE), and mean absolute error (MAE) metrics were used to evaluate the models. For the training data set, the results showed that all four models could predict the splitting tensile strength of SCC made with RA because the R^2^ values for each model had significance higher than 0.75. XG Boost was the model with the best performance, showing the highest R^2^ value of R^2^ = 0.8423, as well as the lowest values of RMSE (=0.0581) and MAE (=0.0443), when compared with the GB, CB, and ETR models. Therefore, XG Boost was considered the best model for predicting the splitting tensile strength of 28-day-old SCC made with RA. Sensitivity analysis revealed that the variable contributing the most to the split tensile strength of this material after 28 days was cement.

## 1. Introduction

Currently, concrete, as a construction material, is in great demand due to the rapid and advanced growth of infrastructure development in many countries, typically utilized in engineered buildings throughout the globe [1,2,3]; this requires the technology surrounding it to permanently change, looking for improvements and innovations. This is why particular types of concrete have recently emerged, such as self-compacting concrete (SCC), representing an acceptable construction potential while also attracting interest in the use of recycled aggregates (RA) [4,5,6,7,8] from construction and demolition waste (CDW) as a substitute to conventional aggregates [9,10,11], minimizing or potentially eliminating the environmental impacts produced by these CDW [12] and allowing the combination of economic development with sustainability and environmental protection [13].

SCC made with RA is one of the most widely used building materials in construction [14,15] due to its compaction characteristics (without mechanical vibration) and its fluidity. It is a high-strength and efficient concrete that guarantees uniformity. However, its complex structure requires a demanding process of mixture design, consisting of cement (Cmt), water (W), mineral admixture (MA), fine aggregates (FA), coarse aggregates (CA), and superplasticizers (SP); this means it is necessary to understand the behavior of it’s mechanical characteristics, such as flexural strength, splitting tensile strength (fst), compressive strength (fsk), modulus of rupture, among other factors [14]. Usually, these properties are identified and measured by performing large-scale experiments, which are typically long, costly, and laborious [3,16]. Therefore, to accurately predict the behavior of these properties, artificial intelligence techniques, such as machine learning (ML), have been employed for their simplicity, reliability, and their ability to learn from experimental data [3,11].

Remarkably, in civil engineering, ML methods have improved the safety, productivity, quality, and maintenance of construction [17,18] and have been used to model and predict the mechanical properties of SCC [16,19,20,21,22]. Therefore, the prediction of these properties through ML saves on the following: laboratory time, waste of concrete components, energy, and cost [3,14,16,20,23,24]. ML can also handle large volumes of data and predict the mechanical properties of SCC with high accuracy [2,3,11].

Among the most widely used ML methods to predict these concrete properties are: decision tree regressor (DTR) [1,25,26,27], random forest (RF) [24,25,28], eXtreme gradient boosting (XG Boost) [29,30], support vector regressor (SVR) [14,21,31], artificial neural network (ANN) [1,22,27,32,33,34,35], and gradient boosting regressor (GBR) [25,29,30]. For example, Lyngdoh et al. [19] employed K-nearest neighbor (KNN), support vector machine (SVM), XG Boost, neural network (NN), least absolute shrinkage, random forest (RF), and selection operator (LASSO) to predict the splitting tensile strength and compressive strength of concrete. Meanwhile, Bui et al. [36] established an expert system based on an artificial neural network (ANN) model and supported by a modified firefly algorithm (MFA) to predict the splitting tensile strength and compressive strength of high-performance concrete. Nguyen et al. [37] used four prediction algorithms: support vector regression (SVR), multilayer perceptron (MLP), gradient boosting regressor (GBR), and eXtreme gradient boosting (XG Boost) to estimate the compression and tensile strength of high-performance concrete. They concluded that the XG Boost and GBR models better predicted the tensile strength and compressive strength of high-performance concrete. Finally, Awoyera et al. [32] modeled several properties of geopolymer self-compacting concrete, namely compressive strength, ultimate strength, and flexural strength, by applying genetic programming techniques (GEP) and artificial neural networks (ANN) and concluded that both GEP and ANN methods yield good predictions from experimental data, with minimal errors.

In particular, splitting tensile strength is one of the mechanical properties of importance in the design of concrete structures [38,39] because cracking in concrete is generally due to tensile stresses that occur under load or due to environmental changes [40]. Machine learning methods have been employed to predict the splitting tensile strength of concrete, with the most widely used being neural networks (ANN) [32,36,41,42,43,44,45,46], support vector machine (SVM) [16,19,37,38,42,44,45,47,48,49], eXtreme gradient boosting (XG Boost) [19,37,44], random forest (RF) [16,19,49], decision tree regressor (DTR) [16,27], gradient boosting regressor (GBR) [16,37], and finally multilayer perceptron (MLPs) [37,49].

### Research Significance

This research aims to compare four machine learning (ML) methods: XG Boost, GB, CB, and ETR, in estimating the splitting tensile strength of 28-day-old SCC made with RA with data obtained from the literature. To the authors’ knowledge, no considerable research has been performed on comparing ML methods on the splitting tensile strength of self-compacting concrete with recycled aggregates, which marks the novelty of the present study. The performance of the ML models was evaluated by R^2^, RMSE, and MAE metrics to determine the most suitable ML algorithm for obtaining reliable, splitting tensile strength predictions.

## 2. Theoretical Background

### 2.1. Machine Learning Methods

**Figure 1:**
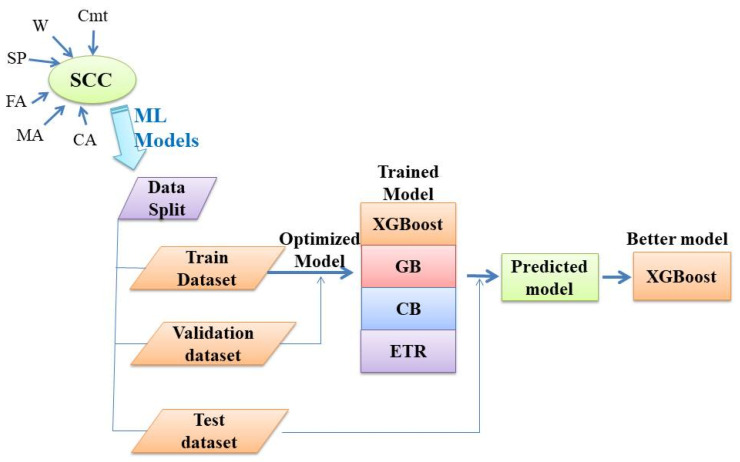
Machine learning process.

ML methods learn from data to then perform classification and prediction. They are becoming more and more popular due to the increasing computational power utilized in the construction sector to estimate the performance of materials [32,37]. The present study applied four ML methods to predict the splitting tensile strength of SCC made with RA: XG Boost, GB, CB, and ETR. These methods were selected based on their extensive usage in related investigations. The ML process is presented in Figure 1. A summary overview of these methods is presented below.

#### 2.1.1. EXtreme Gradient Boosting (XGBoost)

eXtreme gradient boosting (XG Boost) was developed by Chen and Guestrin [50] in 2016 as a scalable, tree-scalable ensemble learning method for tree boosting, helpful for both ML and data mining. XG Boost employs a more regularized formalization of the technique to control overfitting and achieve better performance. As a result, model complexity decreases, and overfitting is largely evaded [51,52]. XG Boost can be employed as an advanced GB method with distributed-parallel processing; this is a result of comparing XG Boost with GB, performed by Chen and Guestrin [50]. In this regard, GB suffers from the drawbacks of overfitting and slowness. Therefore, XG Boost is an ML method that presents two self-compatible regulatory functions (column shrinkage and undersampling), making it more reliable [53].

**Figure 2:**
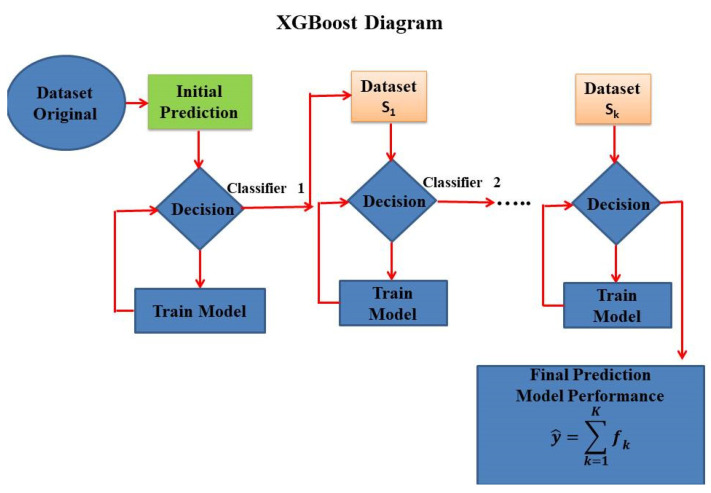
Schematic diagram of XG Boost.

Moreover, it presents better prediction capability, meaning that when there is a large volume of data, the processing time is shorter for XG Boost than for GB. Marani et al. [54] have pointed out that XG Boost employs a regularization function together with a loss function to evaluate the “goodness” of fit of the model. Figure 2 shows the schematic diagram of XG Boost.

#### 2.1.2. Gradient Boosting (GB)

**Figure 3:**
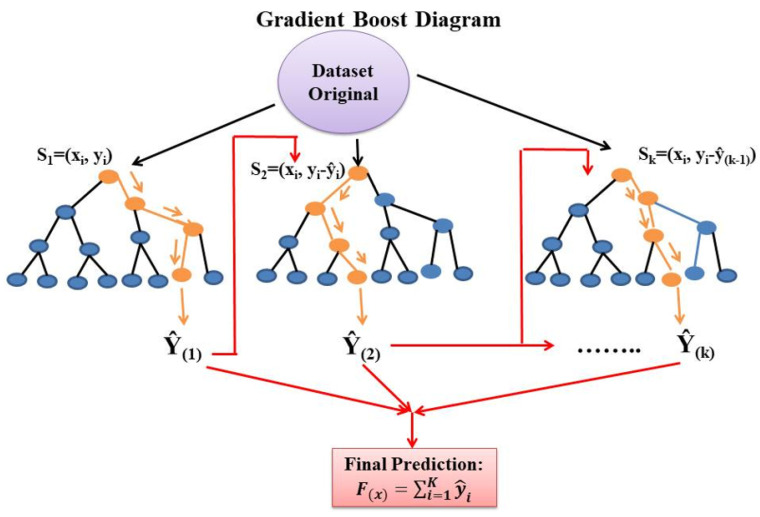
Schematic diagram of Gradient Boost.

Gradient boosting (GB) is a supervised ML method used for both regression and classification problems [54,55]. It was designed in 2001 by Friedman [56] as a method that combines a set of weak models to form a more robust model using additive models. GB connects numerous base learners as a weighted sum to reduce bias and variance, and reweight misclassified data [53,57]. The loss function serves to minimize by employing base learners in boosting iteration [53,57,58]. Several recently developed supervised ML methods, such as XG Boost, LightGBM, and Cat boost, use GB as a basis to improve their ability to adapt to the needs of the moment, improving scalability [57]. Figure 3 shows the schematic diagram of gradient boost.

#### 2.1.3. Cat Boosting (CB)

Cat boosting (CB) is an implementation of GB, proposed by Prokhorenkova et al. [59] that uses binary decision trees as the predictor basis. Two fundamental algorithmic advances introduced in CB were the implementation of ordered boosting (an alternative to the classical algorithm based on permutations) and an innovative algorithm for processing categorical features [59,60]. CB employs one hot max size (OHMS) permutation techniques and object-based statistics focusing on categorical columns [61]. Through the use of the greedy method, tree splitting solves the exponential growth of the combination of features [59]. For each feature that has more categories than OHMS (an input parameter), CB randomly splits (into subsets) the records and converts the labels into integers, and encodes the categorical features by converting them into numbers [61], meaning successful work with categorical features is carried out with the least loss of information [60].

#### 2.1.4. Extra Trees Regressor (ETR)

**Figure 4:**
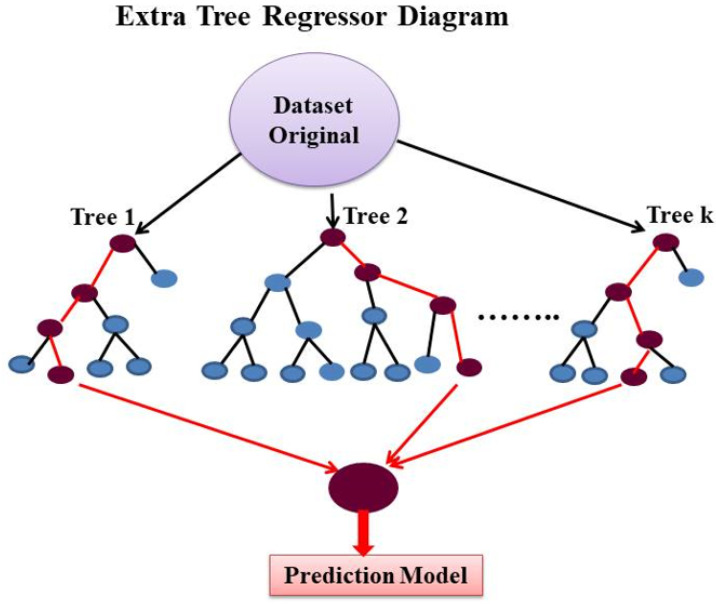
Schematic diagram of Extra Tree Regressor.

Extra trees regressor (ETR) is another supervised ML method proposed by Geurts et al. [62] in 2005, which can be used in regression and classification problems. ETR randomly selects features and cut points by splitting a tree node to train the estimators [62,63,64]. ETR was developed as an extension of GB, employing the same principle [64]. However, it is less likely to overfit a data set [62]. One of the critical differences between these two algorithms is that ETR selects the best aspect and related value to split the node, while GB employs a more discriminative splitting [54]. In addition, ETR, unlike GB, uses the entire training data set to train each regression tree and does not use bootstrapping for training [62,63,64]. Figure 4 shows the schematic diagram of the extra tree regressor.

## 3. Materials and Methods

### 3.1. Experimental Database

The database for this study was made up of 381 samples of SCC made with RA from research articles published in scientific journals, as shown in Table 1. In which the author, the number of mixtures (# mix), and the proportion (% data) contributed to the database are indicated.

**Figure 5:**
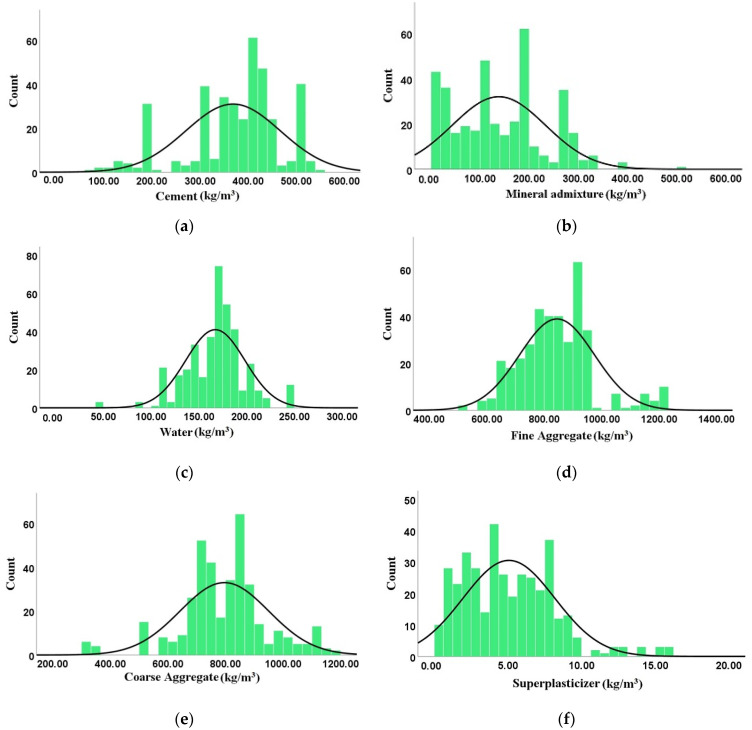
Frequency distribution normal curve of input variables: (**a**) Cement; (**b**) Mineral admixture; (**c**) Water; (**d**) Fine Aggregate; (**e**) Coarse Aggregate; (**f**) Superplasticizer.

From these published papers on the splitting tensile strength of SCC made with RA, Table 2 shows the minimum, maximum, mean, standard deviation, skewness, and kurtosis values of these input variables: Cement (Cmt), Mineral Admixture (MA), Water (W), Fine Aggregate (FA), Coarse Aggregate (CA), Superplasticizer (SP), and Output Splitting Tensile Strength (fst), which were employed to model the splitting tensile strength of SCC made with RA, through the use of ML techniques. In addition, the frequency distribution normal curve of every input variable is displayed in Figure 5, where the behavior of each of the variables can be seen.

### 3.2. Data Pre-Processing

The pre-processing of data is necessary when making data suitable for an ML model. Normalization is a data pre-processing procedure; it eliminates the influence of scales since several features often have different scales and dimensions [92,93]. Normalization ensures that all elements are on the same scale. For this, the data of each part are converted into a number between zero and one; this prevents variables in a higher numerical range from dominating those in a lower numerical range. This process is fundamental to eliminating the influence of a particular dimension and avoiding errors during model development [92,94]. In order to normalize the input and output variables used to model the splitting tensile strength of the SCC made with RA, MaxAbs Scaler was used to scale each character using its maximum value, according to formula (1):

(1) $$x_{scaled}=\frac{x}{max\left(\left|x\right|\right)}$$

where *x* is data.

### 3.3. Data Visualization

**Figure 6:**
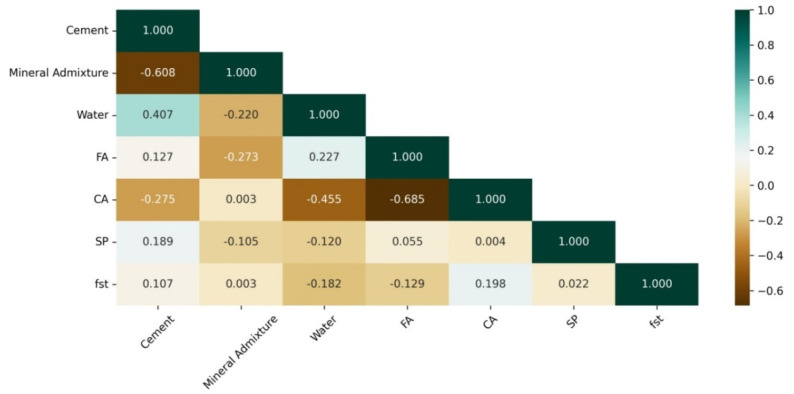
Correlation matrix of the input features.

The correlation between the input characteristics (independent variables) was analyzed to see whether or not there was a dependence between the different parts; this statistical analysis contributes to the optimization of the predictive model [95] because it maximizes the prediction of the results. For this purpose, the Pearson correlation matrix (heat map) was calculated (Figure 6), analyzing the correlation between the independent variables (input variables). Even though there was a relatively high correlation between some of the characteristics, such as mineral admixture and cement (r = −0.608) and coarse aggregates and fine aggregates (r = −0.685), no correlation between the characteristics was higher than 0.80, which indicates that there is no multicollinearity [3,96].

### 3.4. Data Split

To perform the modeling of the 28-day splitting tensile strength of SCC made with RA, a random partition of the data was made within three different sets: training, validation, and test, which helped to evaluate the generalization capacity of the predictive model. The training data set consisted of 267 mixtures (70%), the validation data set consisted of 57 combinations (15%), and the test data set consisted of 57 combinations (15%). Table 3 shows the range and description of the input and output variables for the three data sets.

### 3.5. Model Evaluation

Four metrics were used to evaluate the performance of the models: coefficient of determination (R^2^) (Equation (2)), root mean square error (RMSE) (Equation (3)), and mean absolute error (MAE) (Equation (4)). These metrics estimate errors in the predictions of the splitting tensile strength (of the SCC made with RA after 28 days) when compared with actual observations [9,53,55,97].

(2) $$R^{2}=1-\frac{\sum_{I=1}^{N}{\left(Y_{i}-{\hat{Y}}_{i}\right)}^{2}}{\sum_{i=1}^{n}{\left(y_{i}-{\bar{y}}_{i}\right)}^{2}}$$

(3) $$\mathrm{RMSE}=\sqrt[{2}]{\frac{1}{n}\overset{n}{\underset{i=1}{\sum }}{\left(y_{i}-{\hat{y}}_{i}\right)}^{2}}$$

(4) $$\mathrm{MAE}=\frac{1}{n}\overset{n}{\underset{i=1}{\sum }}\left|y_{i}-{\hat{y}}_{i}\right|$$

where $y_{i}=\mathrm{fst}\;\left(output\;variable\right)$, $\hat{y_{i}}=\mathrm{estimated}$ fst, $\bar{y_{i}}=$ mean experimental fst, and n=number of samples. Currently, the R^2^ value is thought to be the best metric for assessing the model [95,97]. Table 4 shows the range of R^2^ values for prediction model evaluations [54,98,99].

On the other hand, the closer the root mean square error and mean average error values are to zero, the better the ML model’s performance is at predicting the splitting tensile strength of SCC made with RA after 28 days [14,21,55,100].

## 4. Results and Discussions

### 4.1. Comparison of the Predictive Performance of ML Models

Since the R^2^ metric is more intuitive and convenient for comparing the performance of different ML models [95,97], in the following analysis, we adopted it as the primary metric index. Prediction accuracy is reflected in the value of R^2^, and a significant value for this metric indicates that a model has exhibited high prediction accuracy. Values for the RMSE and MAE metrics were also considered; values less than 0.05 indicate that the ML model presents a good fit [95,101] for predicting the splitting tensile strength of 28-day-old SCC made with RA. Table 5 shows the R^2^ results for both the overall data set and the training and test data sets for the models: XG Boost, GB, CB, and ETR. The R^2^ values from the global data set of the four models ranged from 0.7717 to 0.8428 MPa, showing values greater than 0.75. These values indicate that the models have a good predictive capability according to the statistical criteria established for R^2^ [98,99]. Additionally, root mean square error and mean average error values ranged between 0.0225 and 0.0270 MPA and 0.0066 and 0.0078 Mpa, respectively. These values, which are close to zero, indicate that the prediction models XG Boost, GB, CB, and ETR are in high agreement between the predicted data and the actual experimental data obtained from the SCC made with RA.

On the other hand, concerning the training data, it can be seen that the R^2^ values range from 0.9292 to 0.9421 (Table 5), with all values being higher than 0.90; this shows that the four models are good predictors of splitting tensile strength for SCC made with RA.

To select the model of best fit for good predictions of the splitting tensile strength after 28 days (of SCC made with RA), a comparison of the metrics from the test data was made. The XG Boost model had the best predictive performance, with the highest R^2^ value of R^2^ = 0. 8423 (Table 4). Therefore, considering that XG Boost predicts splitting tensile strength with perfect accuracy [98,99], as well as having the lowest RMSE and MAE values (0.0581 MPa and 0.0443 MPa, respectively), indicates that it is a good model fit with high generalizability. According to Guo et al. [44], the high accuracy of the XG Boost model can be attributed to its architecture, which allows for better representation of the relationship between the input and output variables.

**Figure 7:**
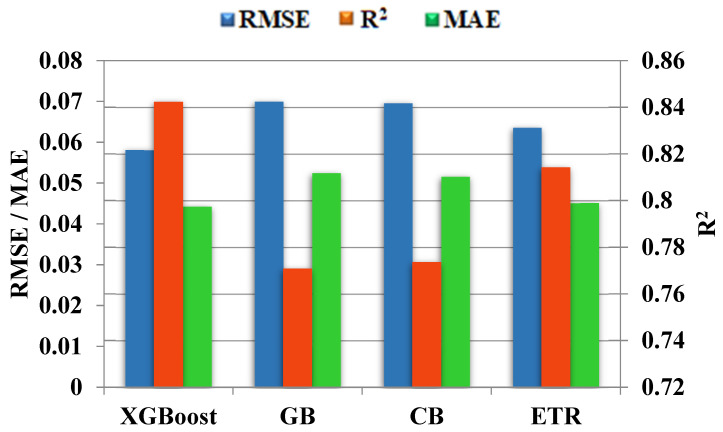
R^2^, RMSE, and MAE of ML models.

Figure 7 shows the predictive behavior of the XG Boost model, with it outperforming the GB, CB, and ETR models with regards to the R^2^ value, as well as having the lowest values for root mean square error and mean average error, which indicates that the XG Boost model presents a good fit for the prediction of 28-day splitting tensile strength in SCC made with RA [19,37,44].

**Figure 8:**
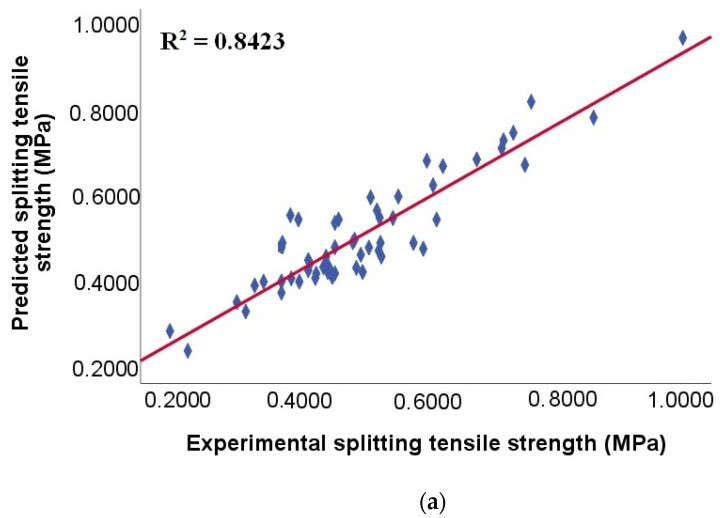

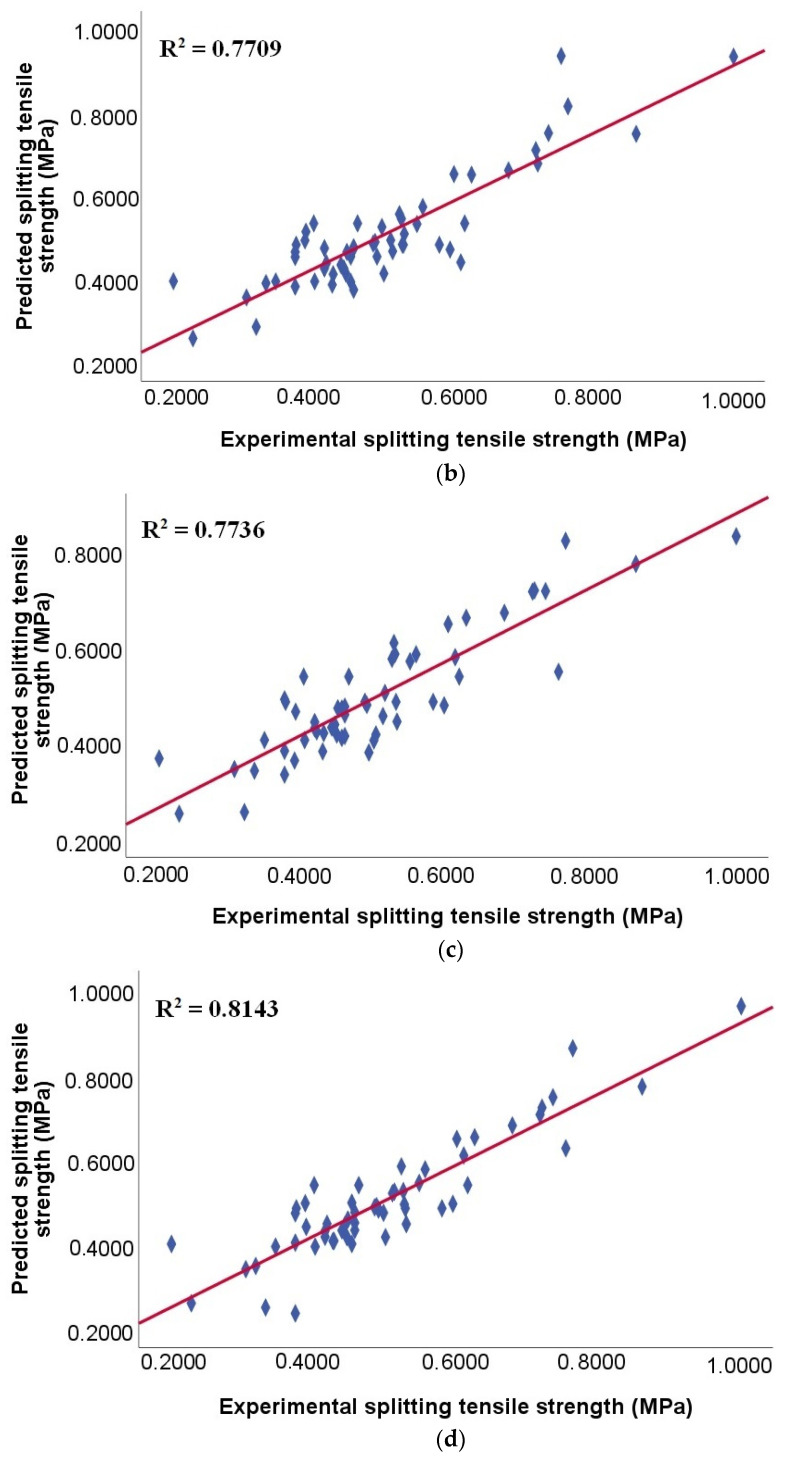
Comparison of the splitting tensile strength for models: (**a**) XG Boost; (**b**) GB; (**c**) CB; and (**d**) ETR, from testing data.

On the other hand, Figure 8 shows the correlation between the experimental and predicted tensile strength for the test data, where it can be seen that all models predict the actual measurements well. However, the scatter plot of the XG Boost model (Figure 5a) has values more closely clustered around the prediction line compared to the other models, thus presenting less scatter. These results show that the XG Boost model made reasonably predictions for splitting tensile strength, similar to findings in previous studies [19,37,44]. In contrast, gradient boost (GB) was the model that showed the lowest accuracy, with an R^2^ value of R^2^= 0.9292 (Table 5) and this is reflected in the scatter plot (Figure 5b), where a higher dispersion of the values around the prediction line is visible. This result agrees with those found by Nguyen et al. [37] when contrasting the importance of XG Boost with gradient boost.

### 4.2. Comparison of the Results of the ML Models

**Figure 9:**
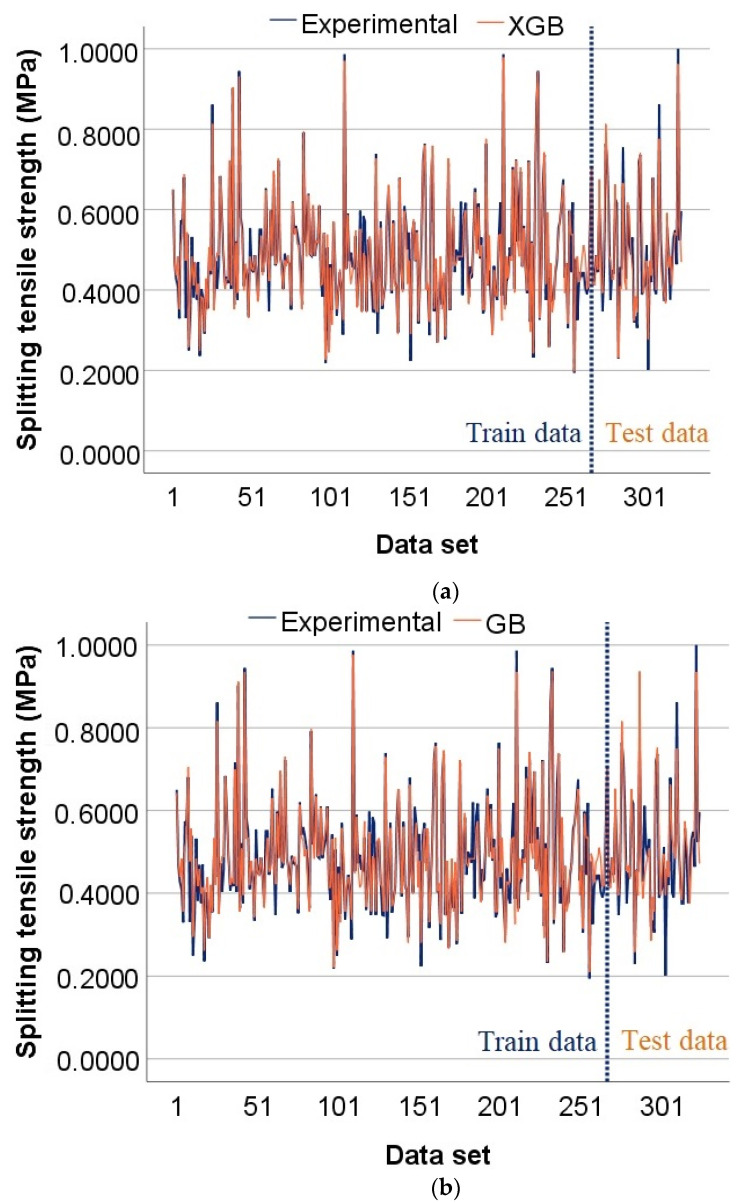

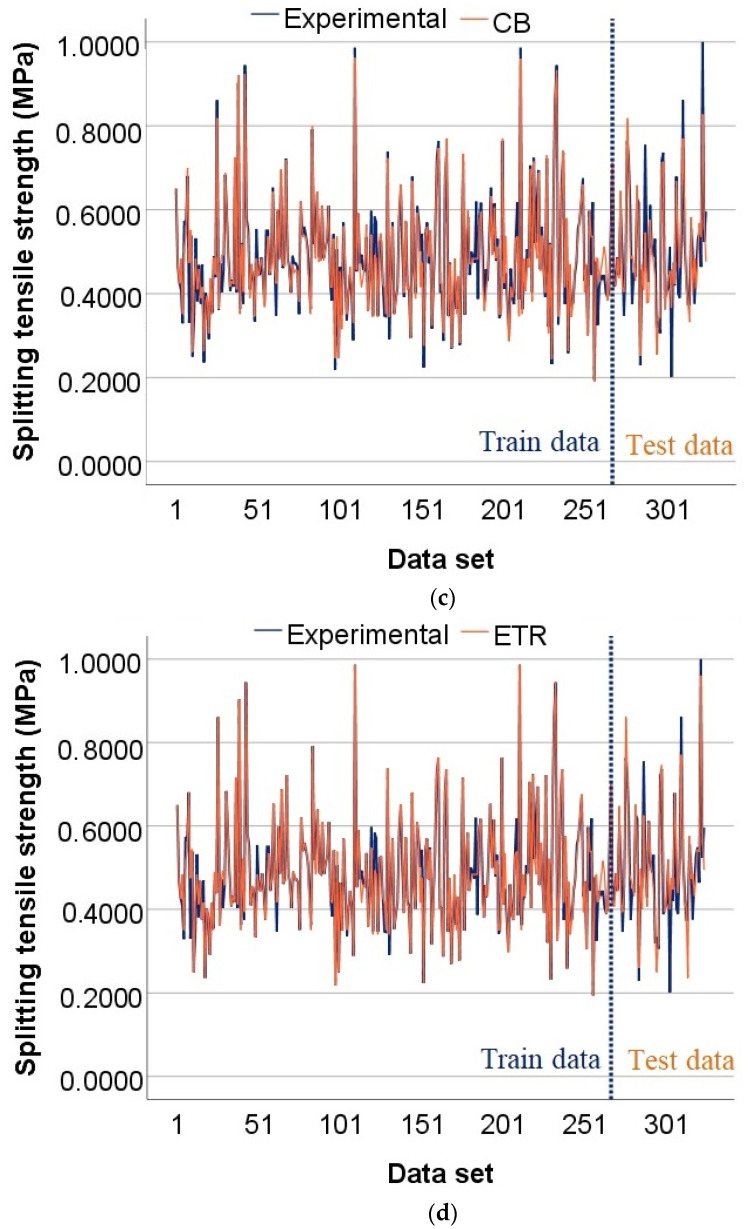
Actual prediction distribution of splitting tensile strength for models: (**a**) XGB; (**b**) GB; (**c**) CB; and (**d**) ETR.

Figure 9 shows the splitting tensile strength of SCC with experimental AR, as predicted by the models XG Boost, GB, CB, and ETR, where the number of samples equal to 267 is the margin of the training and test data results, with the vertical blue stitched line representing this. The given curves illustrate that the values predicted by the XG Boost, GB, CB, and ETR models correlate well with the experimental values of splitting tensile strength. These models allow for the recognition of patterns embedded in the experimental data. The blue colored lines reflect the behavior of the experimental data in each graph, while the red colored lines show the predicted values. The more significant the difference is between the lines of the observed values and the predicted values, the more notable errors have occurred. Thus, the best fitting graph is that of the XG Boost model (Figure 9a). This suggests that the XG Boost model can accurately predict the splitting tensile strength better than GB, CB, and ETR and is therefore the best model.

### 4.3. Sensitivity Analysis

Sensitivity analysis helps to understand the influence of each input variable on the output variables. The higher the sensitivity values, the more significant the impact of the input variables is on the output variable. According to Shang et al. [27], the input variables have a notable effect on the prediction of the output variables. To evaluate the impact of each input variable: cement, mineral admixture, water, fine aggregates, coarse aggregates, and superplasticizers on the uncertainty of the splitting tensile strength (of SCC made with RA), sensitivity analysis was implemented using Equations (5) and (6):

(5) $$S_{i}=\frac{N_{i}}{\sum_{i=1}^{n}N_{i}}*100$$

(6) $$N_{i}=f_{max}\left(x_{i}\right)-f_{min}\left(x_{i}\right),\;i=1,\ldots ,n$$

where $y_{i}=\mathrm{fst}\;\left(output\;variable\right)$, $\hat{y_{i}}=\mathrm{estimated}$ fst, $\bar{y_{i}}=$ mean experimental fst, and n=number of samples.

**Figure 10:**
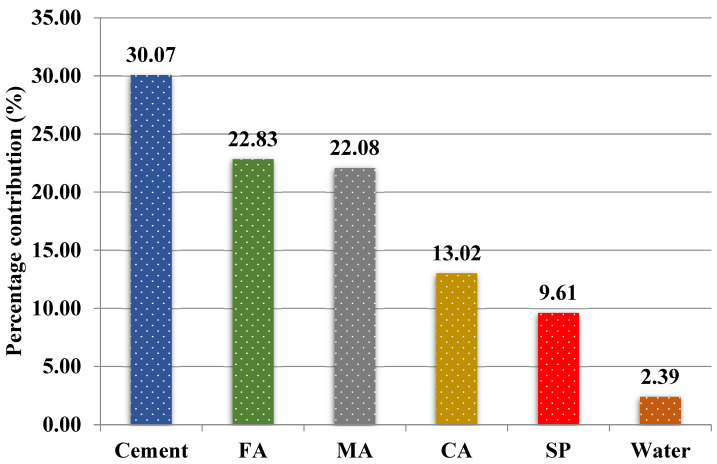
Contributions of input variables toward splitting tensile strength in the XG Boost model. Where FA = fine aggregate, MA = mineral admixture, CA = coarse aggregate, and SP = superplasticizer.

Each of the above input variables plays an essential role in predicting the splitting tensile strength of SCC made with RA, as shown in Figure 10. Cement (30.07%), fine aggregate (22.83%), and mineral admixture (22.08%) made the most significant contributions to the prediction of the fst of SCC made with RA. In relation to this, Shang et al. [27] stated that cement is an element that decisively influences the prediction of the split tensile strength of self-compacting concrete made with RA. It can also be observed that the input variables of coarse aggregate and superplasticizer made similar contributions of 13.02% and 9.61%, respectively. Finally, water (2.39%) was the least influential variable in predicting splitting tensile strength; this result agrees with the findings of previous research [27].

## 5. Conclusions

This study aimed to compare the capacities of four ML methods: XG Boost, GB, CB, and ETR, to predict the splitting tensile strength of 28-day-old SCC made with RA. In addition, the contribution of each input variable in predicting the 28-day splitting tensile strength of SCC made with RA was investigated through sensitivity analysis. For this purpose, the following input variables were implemented: cement, water, mineral admixture, fine aggregates, coarse aggregates, and superplasticizer. To evaluate the predictive capacity of the models, R2, RMSE, and MAE metrics were used. The following conclusions were drawn from this research:

- For the development of the ML models: XG Boost, GB, CB, and ETR, a database of 381 samples from literature published in scientific journals was used. The samples were randomly divided into three data sets: training, validation, and test, each with 267 (70%), 57 (15%), and 57 (15%) samples, respectively.
- The four ML methods predicted the splitting tensile strength of SCC made with RA with satisfactory accuracy; the R^2^ values from the training data for XG Boost, GB, CB, and ETR were 0.9421; 0.9292; 0.9382, and 0.9484, respectively, with all models achieving a value greater than 0.75.
- XG Boost was the best performing model with the highest value of R^2^ (= 0.8423) from the test data set and the lowest values of RMSE (= 0.0581) and MAE (= 0.0443) in comparison with the GB, CB, and ETR models.
- The developed XG Boost model is therefore considered the best for predicting the 28-day splitting tensile strength of SCC made with RA.
- Sensitivity analysis revealed that cement is the input variable that contributes the most (30.07%) to predicting the splitting tensile strength of 28-day-old SCC made with RA. In contrast, water is the parameter that contributes the least (2.39%) towards the same prediction.

## Figures and Tables

**Table 1:** Experimental database.

No	Reference	# Mix	% Data	No	Reference	# Mix	% Data
1	Ali et al., 2012 [65]	18	4.73	22	Nieto et al., 2019 [66]	22	5.78
2	Aslani et al., 2018 [67]	15	3.94	23	Nili et al., 2019 [68]	10	2.63
3	Babalola et al., 2020 [69]	14	3.68	24	Pan et al., 2019 [70]	6	1.57
4	Bahrami et al., 2020 [71]	10	2.63	25	Revathi et al., 2013 [72]	5	1.31
5	Behera et al., 2019 [73]	6	1.57	26	Revilla Cuesta et al., 2020 [74]	5	1.31
6	Chakkamalayath et al., 2020 [75]	6	1.57	27	Sadeghi-Nik et al., 2019 [76]	12	3.15
7	Duan et al., 2020 [77]	10	2.63	28	Señas et al., 2016 [78]	6	1.57
8	Fiol et al., 2018 [79]	12	2.33	29	Sharifi et al., 2013	6	1.57
9	Gesoglu et al., 2015 [80]	24	6.3	30	Sherif and Ali, 2014	15	3.94
10	Grdic et al., 2010 [81]	3	0.79	31	Silva et al., 2016	5	1.31
11	Guneyisi et al., 2014 [82]	5	1.31	32	Singh et al., 2019	12	3.15
12	Guo et al., 2020 [48]	11	2.89	33	Sun et al., 2020	10	2.63
13	Katar et al., 2021 [83]	4	1.05	34	Surendar et al., 2021	7	1.84
14	Khodair et al., 2017 [84]	20	5.25	35	Tang et al., 2016	5	1.31
15	Kou et al., 2009 [85]	13	3.41	36	Thomas et al., 2016	4	1.05
16	Krishna et al., 2018 [86]	5	1.31	37	Tuyan et al., 2014	12	3.15
17	Kumar et al., 2018 [87]	4	1.05	38	Uygunoglu et al., 2014	8	2.10
18	Long et al., 2016 [88]	4	1.05	39	Wang et al., 2020	5	1.31
19	Mahakavi and Chitra, 2019 [89]	25	6.56	40	Yu et al., 2014	3	0.79
20	Manzi et al., 2017 [90]	4	1.05	41	Zhou et al., 2013	6	1.57
21	Martínez-García et al., 2020 [91]	4	1.05		Total	381	100

**Table 2:** Minimum, maximum, mean, standard deviation, skewness, and kurtosis of the input and output variables.

Parameter	Cmt (kg/m^3^)	MA (kg/m^3^)	W (kg/m^3^)	FA (kg/m^3^)	CA (kg/m^3^)	SP (kg/m^3^)	Fst (MPa)
Min ^1^	78.00	0.00	45.50	532.20	328.00	0.00	0.96
Max ^2^	550.00	515.00	246.00	1200.00	1170.00	16.00	7.20
Mean	368.73	138.26	167.29	844.71	796.05	5.07	3.52
SD ^3^	98.38	94.94	31.01	130.52	154.06	3.12	1.00
As ^4^	−0.849	0.396	−0.365	0.593	−0.292	0.852	0.896
K ^5^	0.252	−0.280	1.696	0.728	1.173	1.047	1.477

^1^ Min = minimum value, ^2^ Max = maximum value, ^3^ SD = standard deviation, ^4^ As = skewness, ^5^ K = kurtosis.

**Table 3:** Minimum, maximum, mean, standard deviation, skewness, and kurtosis of input and output variables for each data set.

Data Set	Parameter	Cmt	MA	W	FA	CA	SP	fst
Training	Unit	kg/m^3^	kg/m^3^	kg/m^3^	kg/m^3^	kg/m^3^	kg/m^3^	MPa
	Min ^1^	94.00	0.00	45.50	581.00	328.00	0.00	1.40
	Max ^2^	520.00	390.00	246.00	1200.00	1170.00	16.00	7.10
	Mean	371.83	135.10	168.03	846.72	790.32	4.83	3.51
	SD ^3^	93.32	92.02	31.63	129.38	154.51	2.91	0.99
	As ^4^	−0.91	0.30	−0.20	0.695	−0.53	0.62	0.91
	K ^5^	0.52	−0.68	1.60	0.79	1.35	0.53	0.15
Validation	Min ^1^	78.00	0.00	45.50	532.50	335.00	0.00	0.96
	Max ^2^	520.00	515.00	246.00	1200.00	1170.00	16.00	6.40
	Mean	375.55	143.57	167.53	851.13	789.75	5.86	3.45
	SD ^3^	95.29	107.03	32.34	142.14	151.80	3.40	0.13
	As ^4^	−1.01	0.92	−1.13	0.25	0.01	1.07	0.76
	K ^5^	1.50	1.32	3.21	0.17	1.06	1.50	0.32
Testing	Min ^1^	111.00	0.00	104.30	532.20	530.00	0.00	1.45
	Max ^2^	550.00	320.00	203.40	1200.00	1150.00	16.00	7.20
	Mean	347.36	147.79	163.56	828.85	829.21	5.41	3.61
	SD ^3^	121.12	69.60	26.69	127.79	152.64	3.62	1.06
	As ^4^	−0.43	0.05	−0.57	0.53	0.57	1.07	0.96
	K ^5^	−1.02	−1.14	−0.40	1.55	−0.02	0.89	1.70

^1^ Min = minimum value, ^2^ Max = maximum value, ^3^ SD = standard deviation, ^4^ As = skewness, ^5^ K = kurtosis.

**Table 4:** Statistical criteria for R^2^.

R^2^	Performance Rating	Forecasting Power
≥0.95	Excellent	Very accurate prediction
0.75–0.95	Very good	Prediction good
0.65–0.75	Satisfactory	Predicción acceptable
<0.65	Unsatisfactory	Poor prediction accuracy

**Table 5:** Performance of XB Boost, GB, CB, and ETR with different parameters.

Parameters		XGBoost	GB	CB	ETR
R^2^	Testing	0.8423	0.7709	0.7736	0.8143
	Training	0.9421	0.9292	0.9382	0.9484
	Overall	0.8428	0.7717	0.7744	0.8149
RMSE	Testing	0.0581	0.0700	0.0696	0.0636
	Training	0.0329	0.0365	0.0341	0.0311
	Overall	0.0225	0.0270	0.0269	0.0244
MAE	Testing	0.0443	0.0525	0.0516	0.0451
	Training	0.0188	0.0239	0.0217	0.0127
	Overall	0.0066	0.0078	0.0077	0.0067

## Data Availability

The data are available on request from the corresponding author.
